# Complementary Biomarker Assessment of Components Absorbed from Diet and Creatinine Excretion Rate Reflecting Muscle Mass in Dialysis Patients

**DOI:** 10.3390/nu10121827

**Published:** 2018-11-26

**Authors:** Adrian Post, Akin Ozyilmaz, Ralf Westerhuis, Karin J. R. Ipema, Stephan J. L. Bakker, Casper F. M. Franssen

**Affiliations:** Department of Nephrology, University Medical Center Groningen, University of Groningen, 9713 GZ Groningen, The Netherlands; a.ozyilmaz@umcg.nl (A.O.); r.westerhuis@umcg.nl (R.W.); k.ipema@umcg.nl (K.J.R.I.); s.j.l.bakker@umcg.nl (S.J.L.B.); c.f.m.franssen@umcg.nl (C.F.M.F.)

**Keywords:** dialysis, dietary diaries, excretion, sodium, potassium, phosphate, protein, creatinine, protein energy malnutrition

## Abstract

To prevent protein energy malnutrition (PEM) and accumulation of waste products, dialysis patients require diet adjustments. Dietary intake assessed by self-reported intakes often provides biased information and standard 24-h urinary excretion is inapplicable in dialysis patients. We aimed to assess dietary intake via a complementary, less biased biomarker method, and to compare this to dietary diaries. Additionally, we investigated the prospective association of creatinine excretion rate (CER) reflecting muscle mass with mortality. Complete intradialytic dialysate and interdialytic urinary collections were used to calculate 24-h excretion of protein, sodium, potassium, phosphate and creatinine in 42 chronic dialysis patients and compared with protein, sodium, potassium, and phosphate intake assessed by 5-day dietary diaries. Cox regression analyses were employed to investigate associations of CER with mortality. Mean age was 64 ± 13 years and 52% were male. Complementary biomarker assessed (CBA) and dietary assessed (DA) protein intake were significantly correlated (*r* = 0.610; *p* < 0.001), but there was a constant bias, as dietary diaries overestimated protein intake in most patients. Correlations were found between CBA and DA sodium intake (*r* = 0.297; *p* = 0.056), potassium intake (*r* = 0.312; *p* = 0.047) and phosphate uptake/intake (*r* = 0.409; *p* = 0.008). However, Bland-Altman analysis showed significant proportional bias. During a median follow-up of 26.6 (25.3–31.5) months, nine dialysis patients (23%) died. CER was independently and inversely associated with survival (HR: 0.59 (0.42–0.84); *p* = 0.003). Excretion measurements may be a more reliable assessment of dietary intake in dialysis patients, as this method is relatively free from biases known to exist for self-reported intakes. CER seems to be a promising tool for monitoring PEM.

## 1. Introduction

For hemodialysis patients, managing nutritional intake presents many challenges. While in the general population overnutrition is an increasing problem, in dialysis patients, protein energy malnutrition (PEM) is a major threat. Surveys show that PEM is highly prevalent (25–50%) and is considered the most important risk factor for morbidity and mortality in dialysis patients [1,2,3]. Among the criteria for PEM are loss of muscle mass and insufficient protein intake, and it is for these reasons that dieticians advise a high protein intake in dialysis patients [1]. This advice is, however, complicated by the fact that dialysis patients at the same time require their diet to be restricted in the intake of sodium, potassium and phosphate, to prevent development of volume overload, hypertension, hyperkalemia and hyperphosphatemia, all of which are known to adversely affect prognosis [1,2,4,5,6,7]. The complexity of this need for a high protein intake on the one hand and restrictions on sodium, potassium and phosphate intake on the other makes for difficult dietary advice, where PEM is continuously lurking. To be able to prevent the occurrence of PEM, volume overload, hypertension, hyperkalemia and hyperphosphatemia, there is a great need for methods that allow for objective evaluation of absorbed dietary components. Classic dietary assessment can be done via diaries on dietary intake, but such assessments have many limitations, including underreporting, overreporting, illiteracy and motivation requirements, changes in diet due to self-reflections, errors in portion size estimates, and socially desirable answers [8,9,10]. Additionally, dietary diaries give no information on bioavailability, i.e. the absorbed fraction of the ingested dietary components. In subjects who do not depend on dialysis for renal replacement therapy, the gold standard for unbiased assessment of the intake of several components of the diet, including intake of sodium, potassium and protein, is via measurement of sodium, potassium and urea, respectively, in 24 h urine collections [8,11,12,13,14,15]. For phosphate, assessment via 24 h urine collection provides an estimate of the amount of phosphate absorbed from the gut becoming available for metabolism [16]. 

Since dialysis patients primarily depend on hemodialysis for the removal of water-soluble solutes like sodium, potassium, urea and phosphate, 24 h urinary excretion alone is not a reliable reflection of dietary intake or uptake from the gut. However, if data on 24 h urine collection would be complemented with data on dialysate collected during a dialysis session, this would provide an alternative which is equivalent to 24 h urine collection in subjects who do not depend on dialysis.

Interestingly, with the focus of nutrition in dialysis patients on protein energy malnutrition and preservation of muscle mass, it is relevant to note that assessment of creatinine excretion via 24 h urine collection is considered a reliable method for assessment of muscle mass in subjects not dependent on dialysis, and in previous studies in these subjects we have shown that low creatinine excretion is a very strong predictor of poor long-term outcome, with predictive strength increasing with increasing vulnerability of the population [17,18,19,20,21,22]. 

We aimed to investigate the potential utility of collection of the total dialysate solution during hemodialysis, which is complementary to a complete interdialytic urine collection for providing complementary information on dietary intake and dietary uptake of phosphate from the gut for guidance of dietary advices and for the prevention of PEM and other adverse effects in dialysis patients. We hypothesized that even if dietary intake would be assessed optimally from the perspective of dietary diaries, because of the bias intrinsic to diaries [8,9,10], there would be significant differences and significant bias between the data obtained by means of diaries and the data obtained via the complementary method. 

In this study, we measured 24 h excretion in both dialysate and urine, allowing us to: (1) Set up a complementary biomarker method for assessing 24 h intake of protein, sodium, potassium and phosphate in dialysis patients; (2) to conduct a comparison in 24 h intake between this method and 5-day dietary diaries and (3) to prospectively investigate the association of complementary assessment of creatinine excretion rate (CER) with mortality in dialysis patients. 

## 2. Materials and Methods 

### 2.1. Design and Study Population

This observational study was performed according to ethical standards laid down in the 1964 Declaration of Helsinki and its later amendments, and was approved by the Medical Ethical Committee of the University Medical Center Groningen, The Netherlands. All participating patients gave written informed consent. Inclusion criteria were twice or thrice weekly hemodialysis with 3–5 h per treatment, a dialysis vintage of ≥ 2 months and absence of clinical signs of infection. 

Patients dialyzing three times per week dialyzed on either Monday-Wednesday-Friday or Tuesday-Thursday-Saturday. In both cases the mid-week dialysis session was used in this study. For patients dialyzing twice weekly, the last dialysis session of the week was used. Hypertension was defined as predialysis systolic blood pressure > 140 mmHg and/or diastolic blood pressure > 90 mmHg or use of antihypertensive drugs. A history of diabetes and cardiovascular disease was obtained from the patients’ medical records. Cardiovascular disease was defined as a history of ischemic heart disease, congestive heart failure, coronary artery bypass grafting, percutaneous coronary intervention, stroke, or peripheral vascular disease. Blood pressure and weight were measured before and after hemodialysis. Body surface area (BSA) was calculated using the formula of Du Bois and Du Bois [23].

### 2.2. Dialysis Settings

All studies were performed with the Fresenius 5008 dialysis apparatus with a low-flux dialyzer (Fresenius Medical Care, Bad Homburg, Germany) using smartbag dialysate concentrations (Fresenius Medical Care, Bad Homburg, Germany). Blood flow and dialysate flow were between 200 and 300 mL/min and between 500 and 700 mL/min, respectively. Dialysate temperature was 36.0 or 36.5 °C. Dialysis fluid sodium varied from 136 to 140 mmol/L, potassium from 1 to 3 mmol/L, depending on the plasma potassium concentration, calcium from 1.25 to 1.50 mmol/L and bicarbonate from 34 to 38 mmol/L.

### 2.3. Sample Collection

During the dialysis session, all dialysate was collected in a 200-liter tank. The total dialysate volume was measured by calculating the weight difference of the tank before and after the hemodialysis session. At the end of the dialysis, homogenous samples were taken from the dialysate for analysis. Venous blood was drawn directly from the dialysis line, at the start of hemodialysis and 5 min before the end of the dialysis session. Patients with significant residual diuresis, defined as a urine production of more than 200 mL/24 h, were asked to collect two 24-h urine collections before the hemodialysis session during which the dialysate was collected. For patients with a thrice weekly dialysis schedule this was the complete interdialytic urine production.

### 2.4. Laboratory Measurements

Sodium, potassium, urea, and total protein were measured on Roche routine chemistry analyzers (Modular P/Cobas C, Roche Diagnostics, Mannheim, Germany). Phosphate was measured using a validated ion-exchange chromatography assay with conductivity detection (Metrohm, Herisau, Switzerland). Other laboratory measurements were performed with automated and validated routine methods (Roche Diagnostics, Mannheim, Germany). 

Complementary biomarker assessed (CBA) protein intake was estimated with the Maroni formula, based on a formula equivalent to the formula normally used for estimation of total protein intake based on 24 h urea excretion [24]:

CBA protein intake (g/24 h) = 6.25 × (0.0276 × ((V_Dialysis_ × D_u_ × *n*)/7 + UUE) + 0.031 × BW) + UPE (1)

in which V_Dialysis_ = total volume of dialysate (L); D_u_ = measured urea concentration in the dialysate, which was collected in the tank (mmol/L); *n* = number of dialyses per week; UUE = 24 h urine urea excretion (mmol/24 h), averaged from two 24 h urine collections; BW = body weight (kg); and UPE = 24 h urine protein excretion (g/24 h), averaged from two 24 h urine collections.

Complementary biomarker assessed (CBA) sodium and potassium intake, and CBA phosphate uptake were calculated by combining dialysis and urinary excretion as follows:

CBA sodium intake (mg/24 h) = ((V_Dialysate_ × (D_Na_ – DF_Na_) × *n*) / 7 + UNaE) × 23 (2)

CBA potassium intake (mg/24 h) = ((V_Dialysate_ × (D_K_ – DF_K_) × *n*) / 7 + UKE) × 39 (3)

CBA phosphate uptake (mg/24 h) = ((V_Dialysate_ × D_PO4_ × *n*) / 7 + UPO_4_E) × 95 (4)

in which V_Dialysate_ = total volume of the spent dialysate (L); D_Na_ = measured sodium concentration in the collected dialysate (mmol/L); DF_Na_ = sodium concentration in the dialysis fluid, which flows through the dialysis machine (mmol/L); D_K_ = measured potassium concentration in the collected dialysate (mmol/L); DF_K_ = potassium concentration in the dialysis fluid (mmol/L); D_PO4_ = measured phosphate concentration in the collected dialysate (mmol/L); *n* = number of dialyses per week; UNaE = 24 h urinary sodium excretion (mmol/24 h), averaged from two 24 h urine collections; UKE = 24 h urinary potassium excretion (mmol/24 h), averaged from two 24 h urine collections; and UPO_4_E = 24 h urinary phosphate excretion (mmol/24 h), averaged from two 24 h urine collections.

Creatinine excretion rate (CER) was calculated as follows:

CER (mmol/24 h) = ((V_Dialysis_ × D_Creat_ × *n*) / 7 + UCrE) (5)

in which V_Dialysis_ = total volume of dialysate (L); D_Creat_ = measured creatinine concentration in the collected dialysate (mmol/L); *n* = number of dialyses per week; UCrE = 24 h urinary creatinine excretion (mmol/24 h), averaged from two 24 h urine collections. To account for the difference in muscle mass due to height differences, additional analyses were employed for CER indexed to height (CERH), calculated as follows: CER indexed to height (CERH) = CER / height^2^ [25,26,27,28].

### 2.5. Dietary Intake Assessment

Participating patients were asked to record all their food and fluid intake in a dietary diary for a period of 5 days starting 5 days before the dialysis of interest. The number of servings was expressed in natural units (e.g., slice of bread or apple) or household measures (e.g., cup or spoon). The diaries were self-administered and filled out at home. Dietary data were converted into daily nutrient intakes with the use of the Dutch Food Composition Tables (Nevo 2007 and 2011), using EvryDietist calculating software. Use of phosphate binders and potassium binders were obtained from the patients’ medical records. 

### 2.6. Statistical Analysis

Data analysis and computations were performed with SPSS version 24 (IBM, Armonk, USA), State SE version 15 (StataCorp, Texas, USA), Analyse-IT (Analyse-it Software, Ltd., Leeds, UK) and GraphPad Prism version 5 (GraphPad software, San Diego, USA). Normally distributed data were expressed as mean ± standard deviation (SD), and non-parametric data as median (interquartile range). Categorical variables were expressed as number (%). Pearson correlation coefficients were used to express correlations between complementary biomarker assessed (CBA) intake and dietary assessed (DA) intake. Bland-Altman analyses were used to analyze the bias between CBA and DA intake. In Bland-Altman plots, the difference between CBA intake and DA intake (*y*-axis) were plotted against the mean of CBA intake and DA intake (*x*-axis), where a significant correlation indicates a proportional bias. An absence of proportional bias, but a constant difference among the methods indicates constant bias. A two-sided *p* < 0.05 was considered statistical significance.

The endpoint for prospective analyses was all-cause mortality. Prospective analyses were performed using Kaplan-Meier curves and Cox regression models. Kaplan-Meier curves were made for sex stratified tertiles of CER and CERH. Cox regression analyses were employed to investigate the association of both CER and CERH with all-cause mortality, in which adjustments were made for potential confounders including age, sex, body mass index (BMI), BSA, and systolic blood pressure. Cox regression models were built in a stepwise fashion to avoid overfitting and to keep the number of predictors in proportion to the number of events. 

## 3. Results

### 3.1. Patient Characteristics

CBA and DA intakes were determined in 42 patients, 52% of which were male. Mean age at inclusion was 64 (± 13) years and dialysis vintage 14 (6–45) months. Nearly all patients (41 out of 42) were dialyzed thrice weekly, one patient was dialyzed twice weekly. Most patients (*n* = 34) were dialyzed 4 h per session, while six patients were dialyzed 3 to 3.5 h per session and two patients were dialyzed 4.5 to 5 h per session. Mean BMI and BSA were 25.6 ± 4.3 kg/m^2^ and 1.93 ± 0.21 m^2^, respectively. Hypertension, diabetes and cardiovascular disease were prevalent in 67%, 29% and 43% of the patients, respectively. Half of the patients used phosphate binders (50%). Sevelamer was used by 36% of the patients, calcium carbonate or lanthanum carbonate by 26% and calcium acetate plus magnesium carbonate (OsvaRen) by 10%. Potassium binders were used in 10% of the patients (calcium polystyrene sulfonate in all). An overview of patient characteristics is shown in Table 1.

### 3.2. Clinical and Laboratory Parameters Before and After Dialysis

During dialysis, mean body weight declined from 80.0 ± 16.0 kg before dialysis to 78.7 ± 16.0 kg after dialysis (*p* < 0.001), with a mean ultrafiltration volume of 1926 ± 921 mL. Systolic, but not diastolic blood pressure, declined significantly from 147 ± 21 mmHg to 138 ± 27 mmHg (*p* = 0.012). Plasma potassium (4.9 ± 0.5 vs 3.5 ± 0.4 mmol/L; *p* < 0.001), phosphate (1.6 ± 0.6 vs. 0.8 ± 0.2 mmol/L; *p* < 0.001), urea (20 ± 5 vs. 6 ± 2 mmol/L; *p* < 0.001) and creatinine (707 ± 196 vs. 265 ± 94 µmol/L; *p* < 0.001) all declined during dialysis. Conversely, hemoglobin increased during dialysis (from 6.9 ± 0.7 to 7.4 ± 1.0 mmol/L; *p* < 0.001). The same was true for hematocrit (from 0.34 ± 0.04 to 0.36 ± 0.04%; *p* < 0.001) and plasma albumin (from 40 ± 5 to 43 ± 4 g/L; *p* < 0.001). An overview of clinical and laboratory parameters before and after dialysis is shown in Table 2. 

### 3.3. Comparison of CBA Intake with DA Intake

**Figure 1:**
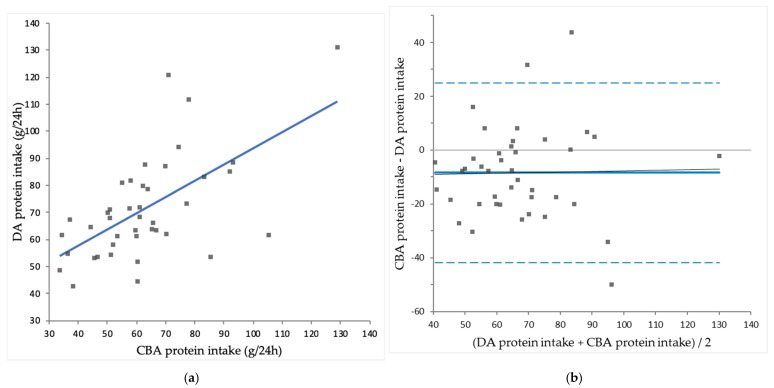
(**a**) Comparison of complementary biomarker assessed (CBA) protein intake and diary assessed (DA) protein intake (*r* = 0.610; *p* < 0.001); (**b**) Bland-Altman plot for CBA protein intake and DA protein intake, showing constant bias (*p* = 0.003), and no proportional bias (*r* = −0.023; *p* = 0.888).

**Figure 2:**
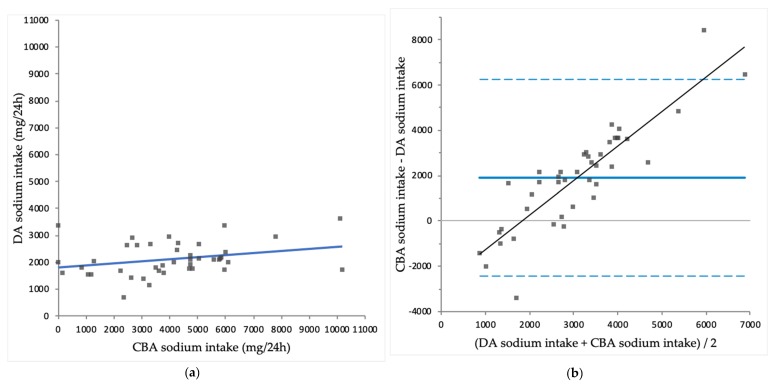
(**a**) Comparison of complementary biomarker assessed (CBA) sodium intake and diary assessed (DA) sodium intake (*r* = 0.297; *p* = 0.056); (**b**) Bland-Altman plot for CBA sodium intake and DA sodium intake, with significant proportional bias (*r* = 0.887; *p* < 0.001).

**Figure 3:**
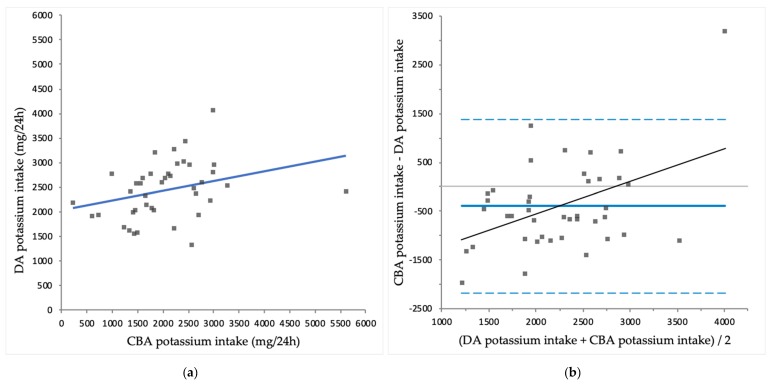
(**a**) Comparison of complementary biomarker assessed (CBA) potassium intake and diary assessed (DA) potassium intake (*r*=0.312; *p* = 0.047); (**b**) Bland-Altman plot for CBA potassium intake and DA potassium intake, with significant proportional bias (*r* = 0.446; *p* = 0.003).

**Figure 4:**
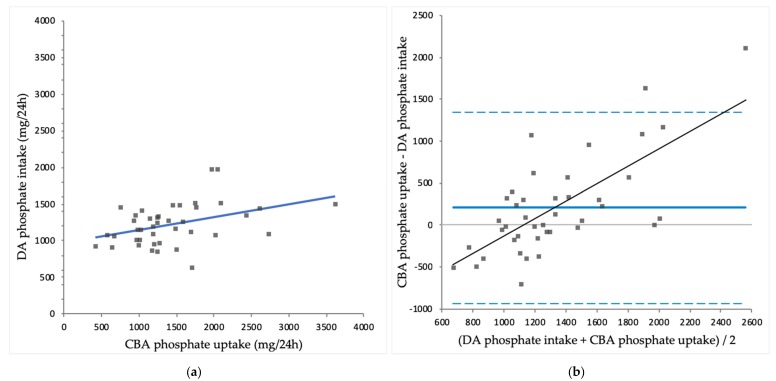
(**a**) Comparison of complementary biomarker assessed (UBA) phosphate uptake and diary assessed (DA) phosphate intake (*r* = 0.409; *p* = 0.008); (**b**) Bland-Altman plot for CBA phosphate uptake and DA phosphate intake, with significant proportional bias (*r* = 0.710; *p* < 0.001).

Mean CBA protein intake was significantly lower than DA protein intake (63 ± 19 vs. 71 ± 19 g/24 h; *p* = 0.003). Mean CBA sodium intake was significantly higher than DA sodium intake (4035 ± 2316 vs. 2123 ± 616 mg/24 h; *p* < 0.001). Mean CBA potassium intake was significantly lower than DA potassium intake (2014 ± 907 vs. 2445 ± 568 mg/24 h; *p* = 0.008). Mean CBA phosphate uptake was significantly higher than DA phosphate intake (1427 ± 637 vs. 1221 ± 276 mg/24 h; *p* = 0.029). An overview of CBA intakes and DA intakes is shown in Table 3. CBA intake is plotted against DA intake for protein (Figure 1a), sodium (Figure 2a), potassium (Figure 3a) and phosphate (Figure 4a).

CBA protein correlated significantly with DA protein intake (*r* = 0.610; *p* < 0.001), while CBA sodium intake did not correlate significantly with DA sodium intake (*r* = 0.297; *p* = 0.056). CBA potassium intake correlated significantly with DA potassium intake (*r* = 0.312; *p* = 0.047) and CBA phosphate uptake correlated significantly with DA phosphate intake (*r* = 0.409; *p* = 0.008). 

The differences between CBA intakes and DA intakes are plotted against the mean of CBA intakes and DA intakes for protein (Figure 1b), sodium (Figure 2b), potassium (Figure 3b) and phosphate (Figure 4b). For protein intake, Bland-Altman analysis showed no proportional bias (*r* = −0.023; *p* = 0.888). However, compared to CBA protein intake, mean DA protein intake was 13% higher and in 30 out of 41 (73%) patients DA protein intake overestimated CBA protein intake, consistent with significant constant bias (*p* = 0.003).

For sodium, potassium and phosphate, Bland-Altman analysis showed proportional bias, as there were significant correlations found between the difference of CBA intakes and DA intakes and the mean of CBA intakes and DA intakes of sodium (*r* = 0.887; *p* < 0.001), potassium (*r* = 0.446; *p* = 0.003) and phosphate (*r* = 0.710; *p* < 0.001). These results indicate that compared to CBA intakes, DA intakes overestimate sodium and potassium intake in the lower end of the intake spectrum, while DA intakes underestimate intakes in the higher end of the intake spectrum. Similarly, compared to CBA phosphate uptake, DA phosphate intake is higher in the lower end of the spectrum, but lower in the higher end of the spectrum of intake and uptake. The differences between methods were largest at the extremes of intake. Exclusion of the four patients who used potassium binders did not have a substantial effect on the correlations for potassium (data not shown).

### 3.4. Prospective Analysis 

**Figure 5:**
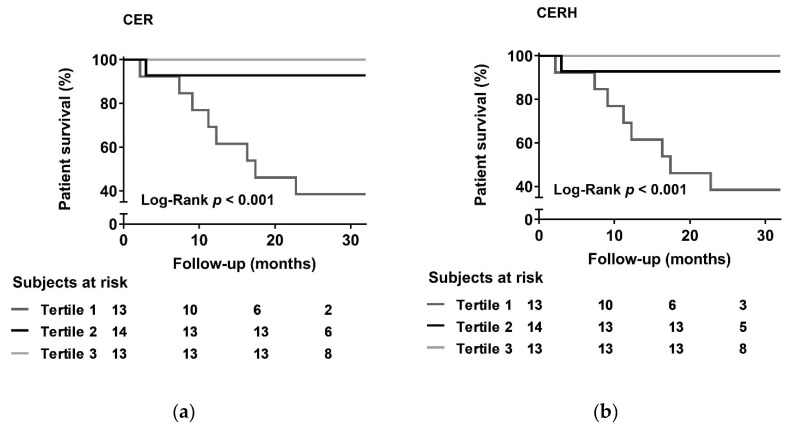
(**a**) Kaplan-Meier curves for sex-stratified tertiles of creatinine excretion rate (CER) and (**b**) creatinine excretion rate, indexed to height^2^ (CERH).

CER and survival data were available for 40 dialysis patients. Mean CER was 8.7 ± 3.3 mmol/24 h and mean CERH was 2.8 ± 1.0 mmol/24 h/m^2^. Median follow-up from baseline was 26.6 (25.3–31.5) months. During this prospective follow-up, nine dialysis patients (23%) died. Patients who died had significantly lower CER (5.8 ± 2.0 vs. 9.6 ± 3.1 mmol/24 h; *p* = 0.001) and CERH (1.8 ± 0.5 vs. 3.1 ± 0.9 mmol/24 h/m^2^; *p* < 0.001) than dialysis patients who survived during follow-up. According to sex-stratified tertiles of CER, the incidence of mortality during follow-up was 8 of 13 (62%) for the lowest tertile, whereas it was 1 of 14 (7%) and 0 of 13 (0%) for the middle and highest CER tertile, respectively (log-rank test *p* < 0.001). Using sex-stratified tertiles of CERH yielded similar results. Kaplan-Meier curves for CER and CERH are shown in Figure 5. Results of Cox regression analyses for mortality are shown in Table 4. CER and CERH were significantly associated with mortality, with hazard ratios of 0.59 (0.42–0.84) (*p* = 0.003) and 0.13 (0.04–0.45) (*p* = 0.001), respectively. These associations remained consistently present, independent of adjustment for potential confounders including age, sex (model 2), BMI (model 3), BSA (model 4) and blood pressure (model 5).

## 4. Discussion

To the best of our knowledge, this study is the first to use urinary and dialysate collections to set up a complementary biomarker method for assessing daily intake of protein, sodium, potassium and intestinal absorption of phosphate in dialysis patients, and to compare this method with dietary intake assessed by 5-day dietary diaries. Analysis of bias showed a constant bias for protein intake, where diaries overestimated protein intake. Compared to CBA intake, mean DA intake was lower for sodium, but higher for potassium. Mean CBA uptake of phosphate was higher than DA phosphate intake. For sodium, potassium and phosphate, significant proportional bias was found. Diaries seemed to overestimate intake in the lower end of the intakes, while underestimating intake in the higher end of intakes, most notably for sodium. Survival analyses showed that CER was a significant predictor of all-cause mortality, with low CER associated with worse survival. 

Diet is a major lifestyle-related risk factor of a wide range of chronic diseases. Contrary to other lifestyle risk factors, dietary intake is more difficult to measure, and inaccurate dietary assessment may be a serious obstacle to understanding the impact of dietary factors on disease. Dietary intake can be assessed through several methods of self-reported dietary intakes, all of which are prone to many potential biases and errors [8,10]. To overcome these potential errors, and the additional bias introduced by the use of food composition tables, recovery biomarkers are often recommended and used to validate self-reported intakes [10,15,29]. To adjust for the removal of solutes by dialysis, we have complemented urinary excretion with dialysis excretion to set up an applicable biomarker method.

Protein energy malnutrition is used to describe a state of decreased body stores of protein and energy fuels (body protein and fat masses) and is also known as protein energy wasting or uremic malnutrition [1]. PEM is a major threat for patients on dialysis, as it is the strongest risk factor for morbidity and mortality in dialysis patients [2]. The precise mechanisms of PEM remain to be elucidated, but the common pathway of potential causes is a state of increased protein degradation and decreased protein synthesis [1,30]. Therefore, the mainstay of preventing PEM is provision of an adequate protein supply. Current Kidney Dialysis Outcome Quality Initiative (KDOQI) guidelines suggest a protein intake of approximately 1.2 grams of protein per kilogram of body weight per day for patients on dialysis [1,31]. A large study (HEMO-study) in 1901 hemodialysis patients used self-reported intake to assess dietary protein intake and showed that these targets were not met [32]. In our study, the KDOQI guidelines would translate to a mean intake of 96 g/24 h, which was not met by either method of assessment. In addition, in most patients, dietary diaries overestimated protein intake, and mean protein intake by dietary diaries was 13% higher than the excretion method. These findings implicate that the true intake of protein in dialysis patients may be lower than the protein intakes found in studies using self-reported intakes, e.g., the HEMO study [32], which may contribute to the high prevalence of PEM in dialysis patients. Diagnostic criteria of PEM can be classified into four categories, biochemistry, body mass, muscle mass and protein intake. In this study, we investigated the potential of muscle mass, as a tool for monitoring protein energy malnutrition in dialysis. Creatinine excretion via 24 h urine collection is considered a reliable method for the assessment of muscle mass in subjects not dependent on dialysis, and in these subjects, low creatinine excretion has been shown to be a very strong predictor of poor long-term outcome [17,18,19,20,21,22]. Similarly, we found a strong association between CER and mortality in this study, despite the low number of participants. CER may therefore serve as a tool for monitoring protein energy malnutrition in dialysis. 

It is important to note that increasing protein intake to prevent PEM also increases the intake of potentially harmful elements, such as sodium, potassium and phosphate. It is therefore also necessary to be able to accurately assess these nutrients. Dialysis patients are given restrictions on sodium intake, as excessive sodium can contribute to hypertension and volume overload, the latter of which can even contribute to PEM [1]. Normally, over 90% of dietary sodium is excreted renally and recovered in urine [33]. Studies comparing dietary records with urinary sodium excretion have found a wide variety of correlation coefficients, ranging from 0.11 among postmenopausal Chinese women with prehypertension to 0.42 in the INTERMAP study, comprising 4680 participants [34,35,36]. Our results lie somewhat in the middle between these, with a correlation of 0.30 between dietary diaries and excretion. Compared to CBA sodium intake, DA sodium intake was nearly 50% lower, even lower if the excretion method was adjusted for a 10% loss of sodium in sweat and feces. Possible reasons for the differences might be underreporting of sodium intake in diaries or the omission to report added salt during cooking or at the table. In addition, we found strong proportional bias among the methods. Compared to the biomarker method, dietary diaries overestimate sodium intake in the lower end of the intake spectrum, while underestimating in the higher end of the intake spectrum. These data fit the possibility of participants giving socially desirable answers, i.e., overreporting when consuming too little and, more relevant, underreporting when consuming too much sodium. Sodium assessment in dialysis patients may therefore be unreliable when using dietary diaries. 

In contrast with the mean underestimation of sodium intake using urinary sodium collection, dietary diaries of potassium intake often exceeded those of urinary collection, which was also seen in our mean data. This is partially because 15–20% of potassium is lost in the feces and another portion is excreted from sweat [37]. Studies comparing dietary diaries with 24 h urinary potassium excretion found a wide range of correlations, varying from 0.23 in 200 adolescents to 0.35 in 149 healthy volunteers [38,39]. Here, a comparable correlation of 0.31 was found between CBA and DA potassium intake. Proportional bias was also found for potassium, though less pronounced. 

Dietary advices for dialysis patients are focused on limiting phosphate intake to prevent hyperphosphatemia as hyperphosphatemia is central to the development and progression of CKD-mineral bone disorder, which is associated with higher fracture, cardiovascular, and mortality risk [40,41]. Under steady state conditions, whole balance phosphate is maintained by adjusting urinary retention of phosphate to match absorbed phosphate from the diet [42]. Therefore, 24 h measurements of urinary phosphate have been considered a reliable biomarker for dietary phosphate absorption. Several studies have confirmed this in patients with preserved kidney function [16,43,44]. In our study we compared CBA phosphate uptake with DA phosphate intake and found that on average CBA phosphate uptake was higher. Since the amount of phosphate that is not absorbed, for instance due to phosphate binders, does not contribute to hyperphosphatemia, phosphate excretion may be a more useful tool to monitor the phosphate load in dialysis patients. Additional reasons that DA phosphate intake may be unreliable is the dependence on nutrient databases, which are often inaccurate or incomplete for phosphate. Direct chemical analysis of foods showed that databases can drastically underestimate phosphate content, up to 70% [45,46,47,48,49]. 

The strength of this study is that we collected the total dialysate instead of taking several samples during the dialysis sessions, thereby increasing the accuracy of the dialysate excretion measurements. Also, dietary diaries were recorded for 5 days, decreasing the possible bias due to day-to-day variability in food intake. However, there are also some limitations, for instance the relatively small sample size of the study. A second limitation is that CER was only measured at one point in time, while multiple measurements could have allowed us to calculate CER change over time, which could be more reliable. It should also be noted that we presented CER as a tool for monitoring PEM, but diagnosis and follow-up of PEM should be based on multiple assessment tools, preferably in at least three out of the four earlier mentioned categories, biochemistry, body mass, muscle mass, and protein intake [1]. In addition, we did not assess muscle mass by other methods, including magnetic resonance imaging, computed tomography, dual-energy *X*-ray absorptiometry and bioelectric impedance analysis [50], which precluded us from comparing the present method with these techniques. Furthermore, because our participants were Caucasian, our results cannot be extrapolated to other ethnic groups. Most importantly, we have assumed that our excretion method for dialysis patients is the equivalent of 24 h urinary excretion in a non-dialyzing setting. While this is theoretically the case, it is still necessary to first assess its validity, reproducibility, and ability to assess the variation between different dialysis sessions. 

## 5. Conclusions

In conclusion, combined urinary and dialysate collection have been used to set up an unbiased biomarker method for assessing the dietary intake of protein, sodium, potassium and phosphate. We have shown that even if nutritional intake is assessed optimally from the perspective of diaries, e.g., 5-day dietary diaries, there are significant differences and bias between the data obtained by means of the biomarker method and the data obtained by dietary diaries. A significant correlation was found between the methods for assessing protein intake, however, dietary diaries overestimated protein intake in most patients. These findings implicate that the true intake of protein in dialysis patients may be lower than the protein intakes found in studies using self-reported intakes, which is worrisome in the light of the highly prevalent PEM in dialysis patients. Survival analysis showed that creatinine excretion rate could be of interest as a tool for monitoring PEM in dialysis patients. Additionally, for sodium, potassium and phosphate, significant proportional bias was found between both methods. Compared to CBA intake, DA intakes overestimated intake in the lower end of the intakes, while underestimating intake in the higher end of intakes, most notably for sodium. Dialysate and urinary excretion measurements may serve as a more reliable assessment of dietary intake in dialysis patients, as this method is free from the many biases known to self-reported dietary intakes, and may be used as a tool for guiding dietary interventions, using multiple measurements. However, larger studies are warranted to assess the validity, reproducibility, sensitivity and value of this method. 

## Figures and Tables

**Table 1:** Patient characteristics.

Baseline Characteristics	Average/Number	Range
**Demographics**		
Age, years	64 ± 13	25–86
Gender, *n* male (%)	22 (52)	
Race, *n* Caucasian (%)	39 (93)	
**Dialysis-related**		
Dialysis sessions, n (%)		
2 sessions per week	1 (2)	
3 sessions per week	41 (98)	
Hours per dialysis, n (%)		
3 to 3.5 h	6 (14)	
4 h	34 (81)	
4.5 to 5 h	2 (5)	
Residual diuresis, *n* (%)	22 (52)	
Urinary volume, L	0.84 ± 0.57	0.14–2.39
Dialysis vintage, months	14 (6–45)	2–202
Ultrafiltration volume, ml	1926 ± 921	1425–2725
**Body composition**		
Target body weight, kg	80.2 ± 15.6	72.5–89.9
Interdialytic weight gain, kg	1.17 ± 1.12	−1.7–4.4
Height, m	1.75 ± 0.09	1.66–1.83
BMI, kg/m^2^	25.6 ± 4.3	22.7–28.8
BSA, m^2^	1.93 ± 0.21	1.82–2.06
**Pre-existing disease**		
Hypertension, *n* (%)	28 (67)	
Diabetes, *n* (%)	12 (29)	
Cardiovascular disease, *n* (%)	18 (43)	
**Medication usage**		
Use of phosphate binders, *n* (%)	21 (50)	
Sevelamer	15 (36)	
Calciumcarbonate or lanthanumcarbonate	11 (26)	
Calciumacetate and magnesiumcarbonate (OsvaRen)	6 (14)	
Use of potassium binders, *n* (%) *	4 (10)	

* Used potassium binder was calcium polystyrene sulfonate. Abbreviations: BMI, body mass index; BSA, body surface area.

**Table 2:** Clinical and laboratory parameters before and after dialysis.

Variable	Before Dialysis	After Dialysis	*p*-Value
Clinical			
Systolic blood pressure (mmHg)	147 ± 21	138 ± 27	0.012
Diastolic blood pressure, (mmHg)	69 ± 12	67 ± 11	0.163
Pulse, min^−1^	74 ± 14	73 ± 12	0.631
Body weight, kg	80.0 ± 16.0	78.7 ± 16.0	< 0.001
Laboratory			
Hemoglobin (mmol/L)	6.9 ± 0.7	7.4 ± 1.0	0.001
Hematocrit	0.34 ± 0.04	0.36 ± 0.04	0.012
Sodium (mmol/L)	138 ± 3	139 ± 2	0.023
Potassium (mmol/L)	4.9 ± 0.5	3.5 ± 0.4	< 0.001
Phosphate (mmol/L)	1.6 ± 0.6	0.8 ± 0.2	< 0.001
Albumin (g/L)	40 ± 5	43 ± 4	< 0.001
Urea (mmol/L)	20 ± 5	6 ± 2	< 0.001
Creatinine (µmol/L)	707 ± 196	265 ± 94	< 0.001

**Table 3:** Mean values of complementary biomarker assessed (CBA) intakes and dietary assessed (DA) intakes of protein, sodium, potassium and phosphate.

Variable	CBA Intake *	DA Intake	*p*-Value
Protein (g/24 h)	63 ± 19	71 ± 19	0.003
Sodium (mg/24 h)	4035 ± 2316	2123 ± 616	< 0.001
Potassium (mg/24 h)	2041 ± 907	2445 ± 568	0.008
Phosphate (mg/24 h)	1427 ± 637	1221 ± 276	0.029

* = uptake in the case of phosphate.

**Table 4:** Cox regression analyses for prediction of patient mortality based on excretion of creatinine excretion rate (CER) and creatinine excretion indexed to height^2^ (CERH).

Model	CER		CERH	
	HR (95% CI)	*p*-Value	HR (95% CI)	*p*-Value
1	0.59 (0.42–0.84)	0.003	0.13 (0.04–0.45)	0.001
2	0.50 (0.29–0.83)	0.007	0.14 (0.03–0.61)	0.009
3	0.47 (0.28–0.79)	0.005	0.12 (0.03–0.56)	0.007
4	0.49 (0.29–0.82)	0.007	0.14 (0.03–0.62)	0.010
5	0.50 (0.30–0.82)	0.007	0.14 (0.03–0.61)	0.009

Model 1, crude model; model 2, adjusted for age and sex; model 3, as model 2, additionally adjusted BMI; model 4, as model 2, additionally adjusted for BSA; model 5, as model 2, additionally adjusted for systolic blood pressure.
